# Indents

**Published:** 1902-11-15

**Authors:** 


### INDENTS.

☞ **Brief Articles of Technical or Scientific Value will receive notice and comment. Contributions invited.**

Caroid as a Pulp Digestor.—Dr. L. Greenbaum in a paper on root fillings in the September *Cosmos*, calls attention to the use of caroid for treatment of pulp canals from which it is difficult to remove the pulp by instrumentation or otherwise. Caroid is a vegetable digestant made by mixing *carica papaya* and *colza*. A saturated solution is on the market under the name of "*Caroid Solvent*." It is used as a pulp digestant by introducing a few drops into the pulp chamber by means of a pipette and sealing it in place for two or three days with a temporary stopping, when it can be removed by irrigation with an alkaline solution. It is non-irritant and penetrates the canals thoroughly. It acts independently of its environment, temperature or changing media.

Hydrogen Dioxid and Its Misuse.—With reference to the use of hydrogen dioxid, I do not think it should ever be used in any enclosed cavity unless you have two points of egress for it. You are familiar with its expansibility, and on that account if you inject it into an enclosed cavity, as I have done it once, you must have plenty of egress for it. Were I to come across a case of extensive pus formation, I would first try and remove as much of it as possible with an antiseptic wash before I would use hydrozone or any peroxid of hydrogen preparations. The essayist spoke of using a saturated solution of boracic acid, which no doubt is very good, but something else added would be better than boracic acid alone. Dr. Freeman has a preparation that he calls borate of cassia. It can be used nicely in such cases. It is a cleansing preparation and a good germicide.—Dr. Pruyn, in *Review.*

Skill Required for Cleaning Teeth.—The placing of the teeth and adjacent parts in a perfectly clean and healthy condition requires more real skill and practical knowledge than the placing in of a filling. This statement may be regarded as an exaggeration, but it is certain that a careful consideration of all that this operation requires will convince the thoughtful mind that this is not an overstatement. Filling teeth is purely a mechanical operation, and, the foundation principles once acquired, it can be carried on continuously upon the same lines by one of ordinary mechanical ability. To clean teeth and place the adjacent structures in a normal healthy condition means not only an expert ability in the use of instruments, but a quite thorough knowledge of special pathology and therapeutics, and this can be only acquired by long months, and it may be years, of training.—*International Dental Journal.*

Amalgam Filling.—In an article in the May *Dental Summary*, Dr. E. K. Wedelstaedt makes the following sensible comments:

"It is just as essential that an amalgam filling should be trimmed and polished as it is that the cavity in the tooth should be filled. From the number of unfinished fillings I see I can not help saying that there are too many men who charge simply for making the filling. They seem to forget that more work and skill are required to properly finish a filling than are necessary in preparing the cavity and making the filling. Altogether too little attention is given this subject of finishing fillings.

"After an alloy has been mixed with mercury, been well kneaded and is ready for the cavity, the great majority of practitioners believe that how it is placed in that cavity makes little difference. This is a mistake that leads directly to failure, for compressing the amalgam and the use of instruments are both factors that must receive most careful consideration, provided we desire to get the best results from the use of amalgam.

"If amalgam must be used in a cavity, remember that the best results are obtained when attention is given to proper amalgamation, strongly compressing the amalgam, and to instrumentation. If there is an unwillingness on the part of the operator to follow certain well-known rules for using amalgam, some other filling material had better be placed in that cavity."

Javanese Method of Narcosis.—L. Steiner describes in the *Arch. f. Schiffs- u. Tropen-Hygiene, v. 12*, a method of narcosis which has been long practiced in Java. The hands are placed on the neck of the subject, the fingers meeting at the back, and the carotid artery is briefly compressed with the tumbs, back of and a trifle below the lower jaw. The artery is pressed back toward the spine. Only 5 out of 30 subjects failed to respond to his application of this maneuver. The head falls back and the subject seems to be in a profound slumber, from which he awakes in a few minutes as if suddenly aroused. The effect can not be due to suggestion, as the same maneuver avoiding the arteries fails to produce any effect. The procedure is called by a Javanese term which signifies "compression of the sleep vessel." The popular name for the carotid artery in Russian, by the way, is also the "sleep artery;" and "carotid" is derived from the Greek *karos*, sleep. He has never witnessed or heard of any accidents from this method of narcosis, which is widely practiced on the island, frequently associated with general massage. The patients do not vomit, and there is no incontinence of urine or feces. He opened an inguinal abscess in one case while the patient was unconscious. He is inclined to advocate this absolutely harmless method of narcosis as worthy of a place in surgery, on account of the rapidity with which it can be accomplished and the rapid awakening. The procedure may also prove effective in combating cephalalgia, vertigo and insomnia.—*Digest*.

A Severe Case of Carbolic Acid Gangrene.—There is reason, according to the observation of Sheldon (*Medical Record*, April 5, 1902), to believe that the idiosyncrasy of the patient and the surrounding conditions play a part in carbolic acid gangrene, and that such gangrene may extend beyond the borders of the area brought in contact with the acid. Two cases of gangrene following the local application of acid have been reported by Fisher, and the patients were brothers. Sheldon's first case was reported over a year ago. His second case was that of a woman, twenty-six years old, unmarried, with a negative history. She had applied a few drops of fifty percent, solution of carbolic acid to a corn on the outer side of the second toe of the left foot for about half a minute, and the whole foot was then washed with water. Twelve hours later burning was felt over the entire foot, the affected toe was reddened and slightly swollen, except where the acid had been applied, which area was black and felt quite "dead." Destruction of tissue continued for five days. At the end of the first week the symptoms gradually abated, the dead skin sloughed, the redness disappeared, and evidences of resolution could be noted. The healing was slow and resulted in the forming of considerable amount of scar tissue. The treatment consisted in keeping the foot clean. During the progressive stage a dilute solution of cocain was occasionally applied to the foot to relieve pain. Two hypodermic injections of morphin were given for the same purpose. When resolution began, zinc oxid ointment and Lassar's paste were used locally. Two years prior to this case, the patient and her sister accidentally got some carbolic acid solution, strength unknown, on their fingers. The two fingers of the patient and a finger and thumb of the sister were affected somewhat as the patient's foot was affected. There was pain, burning, itching, and redness, with gangrene of the skin, followed by sloughing and resolution.—*Therapeutic Gazette.*

